# VaximmutorDB: A Web-Based Vaccine Immune Factor Database and Its Application for Understanding Vaccine-Induced Immune Mechanisms

**DOI:** 10.3389/fimmu.2021.639491

**Published:** 2021-03-12

**Authors:** Kimberly Berke, Peter Sun, Edison Ong, Nasim Sanati, Anthony Huffman, Timothy Brunson, Fred Loney, Joseph Ostrow, Rebecca Racz, Bin Zhao, Zuoshuang Xiang, Anna Maria Masci, Jie Zheng, Guanming Wu, Yongqun He

**Affiliations:** ^1^College of Literature, Science, and the Arts, University of Michigan, Ann Arbor, MI, United States; ^2^Central Michigan College of Medicine, Mt. Pleasant, MI, United States; ^3^Department of Computational Medicine and Biology, University of Michigan, Ann Arbor, MI, United States; ^4^Department of Medical Informatics and Clinical Epidemiology, Oregon Health and Science University, Portland, OR, United States; ^5^Department of Biostatistics and Bioinformatics, Duke University School of Medicine, Durham, NC, United States; ^6^Department of Genetics, University of Pennsylvania Perelman School of Medicine, Philadelphia, PA, United States; ^7^Unit for Laboratory Animal Medicine, University of Michigan Medical School, Ann Arbor, MI, United States; ^8^Department of Microbiology and Immunology, University of Michigan Medical School, Ann Arbor, MI, United States; ^9^Center for Computational Medicine and Biology, University of Michigan, Ann Arbor, MI, United States

**Keywords:** vaccine, vaccine immune factor, immunology, database, bioinformatics, Reactome pathways

## Abstract

Vaccines stimulate various immune factors critical to protective immune responses. However, a comprehensive picture of vaccine-induced immune factors and pathways have not been systematically collected and analyzed. To address this issue, we developed VaximmutorDB, a web-based database system of vaccine immune factors (abbreviated as “vaximmutors”) manually curated from peer-reviewed articles. VaximmutorDB currently stores 1,740 vaccine immune factors from 13 host species (e.g., human, mouse, and pig). These vaximmutors were induced by 154 vaccines for 46 pathogens. Top 10 vaximmutors include three antibodies (IgG, IgG2a and IgG1), Th1 immune factors (IFN-γ and IL-2), Th2 immune factors (IL-4 and IL-6), TNF-α, CASP-1, and TLR8. Many enriched host processes (e.g., stimulatory C-type lectin receptor signaling pathway, SRP-dependent cotranslational protein targeting to membrane) and cellular components (e.g., extracellular exosome, nucleoplasm) by all the vaximmutors were identified. Using influenza as a model, live attenuated and killed inactivated influenza vaccines stimulate many shared pathways such as signaling of many interleukins (including IL-1, IL-4, IL-6, IL-13, IL-20, and IL-27), interferon signaling, MARK1 activation, and neutrophil degranulation. However, they also present their unique response patterns. While live attenuated influenza vaccine FluMist induced significant signal transduction responses, killed inactivated influenza vaccine Fluarix induced significant metabolism of protein responses. Two different Yellow Fever vaccine (YF-Vax) studies resulted in overlapping gene lists; however, they shared more portions of pathways than gene lists. Interestingly, live attenuated YF-Vax simulates significant metabolism of protein responses, which was similar to the pattern induced by killed inactivated Fluarix. A user-friendly web interface was generated to access, browse and search the VaximmutorDB database information. As the first web-based database of vaccine immune factors, VaximmutorDB provides systematical collection, standardization, storage, and analysis of experimentally verified vaccine immune factors, supporting better understanding of protective vaccine immunity.

## Introduction

As one of the most influential inventions in modern medicine, vaccines stimulate various immune responses in the host's body to protect it from the antigen. The common vaccine-induced immune pathways include innate responses, antigen processing and presentation, adaptive T and B cell responses, and their specific regulations (1). The innate immune system senses microbes through pattern-recognition receptors (PRRs) in various cells such as dendritic cells and macrophages (2). Important PRRs include Toll-like receptors (TLRs) (3), C-type lectin-like receptors (4), and cytosolic Nod-like and RIG-I-like receptors (5), which can sense various microbial stimuli. After antigen presentation, two types of adaptive immune responses can be induced: humoral response and cell-mediated immunity. The humoral response is primarily mediated by antibodies that are generated by B-cells and target specific areas of antigens. In contrast, cell-mediated immunity primarily involves the activation and production of T helper cells, cytotoxic T lymphocytes, macrophages, natural killer cells, and cytokines in response to antigens (6). It has also been hypothesized that vaccine-induced immune responses may have their specific immune patterns different from those of natural virulent pathogen infections (7).

Various immune factors have been identified to be critical to vaccine-induced protective immune responses against pathogens. The importance of vaccine immune factors is clear when certain immunodeficiencies exist and protective immunity against various pathogens cannot be attained through vaccination. For example, Bruton's agammaglobulinemia is a disease which leads a lack of B cell maturation causing hypogammaglobinemia of all classes of immunoglobulin. Since the vaccine cannot induce these immune factors (immunoglobulin), a protective response cannot be formed (8). This same issue can be seen in other immunodeficiencies such as severe combined immunodeficiency and common variable immunodeficiency. These diseases reveal the importance of a host's response to the vaccine and how individual biology can influence vaccine efficacy.

In this study, we hypothesize that different experimental and biological conditions would change vaccine-induced immune responses, which are mediated by different immune factors. The immune factor is defined as an immune-related host gene or gene-derived product such as protein in response to a specific stimulus such as vaccine. After the administration of a vaccine, an immune factor is activated to regulate and modulate the appropriate immune response in the host. Given different experimental conditions, vaccine-induced immune factors (abbreviated as “vaximmutor” in this paper) may have different profiles. The conditions may include vaccine formulation, pathogen, and vaccination route, etc. The study of vaximmutors is essential to understand immune responses of the vaccines. Understanding the similarities and differences between vaccines/vaximmutors under various experimental conditions can help decipher the biological mechanisms behind vaccine immunity and support advanced rational vaccine development. It has been shown that intrinsic factors specific to the host, such as age, genetics, sex, and comorbidities, and vaccine related factors, like adjuvants, vaccine schedule, and the specific type, strongly influence the response to a vaccine (9). We aim to look at the influence of experimental conditions and vaccine related factors through an in-depth look at the immune response elicited from vaccines.

This article reports VaximmutorDB (http://www.violinet.org/vaximmutordb), a web-based database that focuses on the collection, storage, annotation, and analysis of vaccine-induced immune factors. VaximmutorDB is a database under the umbrella of the VIOLIN (http://www.violinet.org), a web-based comprehensive vaccine database and analysis system (10, 11). VaximmutorDB also links various vaximmutors to different vaccine types, formulations, and conditions reported in the other parts of the VIOLIN database. By collecting and analyzing the vaximmutors and their related vaccines and experimental conditions, we were able to identify new insights into the mechanisms of vaccine-induced immune responses.

## Methods

### System and Database Design

The VaximmutorDB web application (http://www.violinet.org/vaximmutordb) was supported by the Apache HTTP server hosted at two HP ProLiant DL380 G6 servers computers that run the RedHat Enterprise Linux operating system (release 5.8, https://www.redhat.com/). A MariaDB database (version 5.5.64, https://mariadb.org/) server that stores data and a PHP web application server that presents HTML-based web pages for users to view in the browser are also installed at these two computers. The web page includes many functions such as vaximmutor data query and BLAST sequence similarity analysis. The development of these features was achieved using the PHP programming language (version 7.2.15, https://www.php.net) and scripts that interact with the internal MariaDB relational database.

### Semi-Automatic Data Annotation, Submission, Review, and Approval

**Figure 1:**
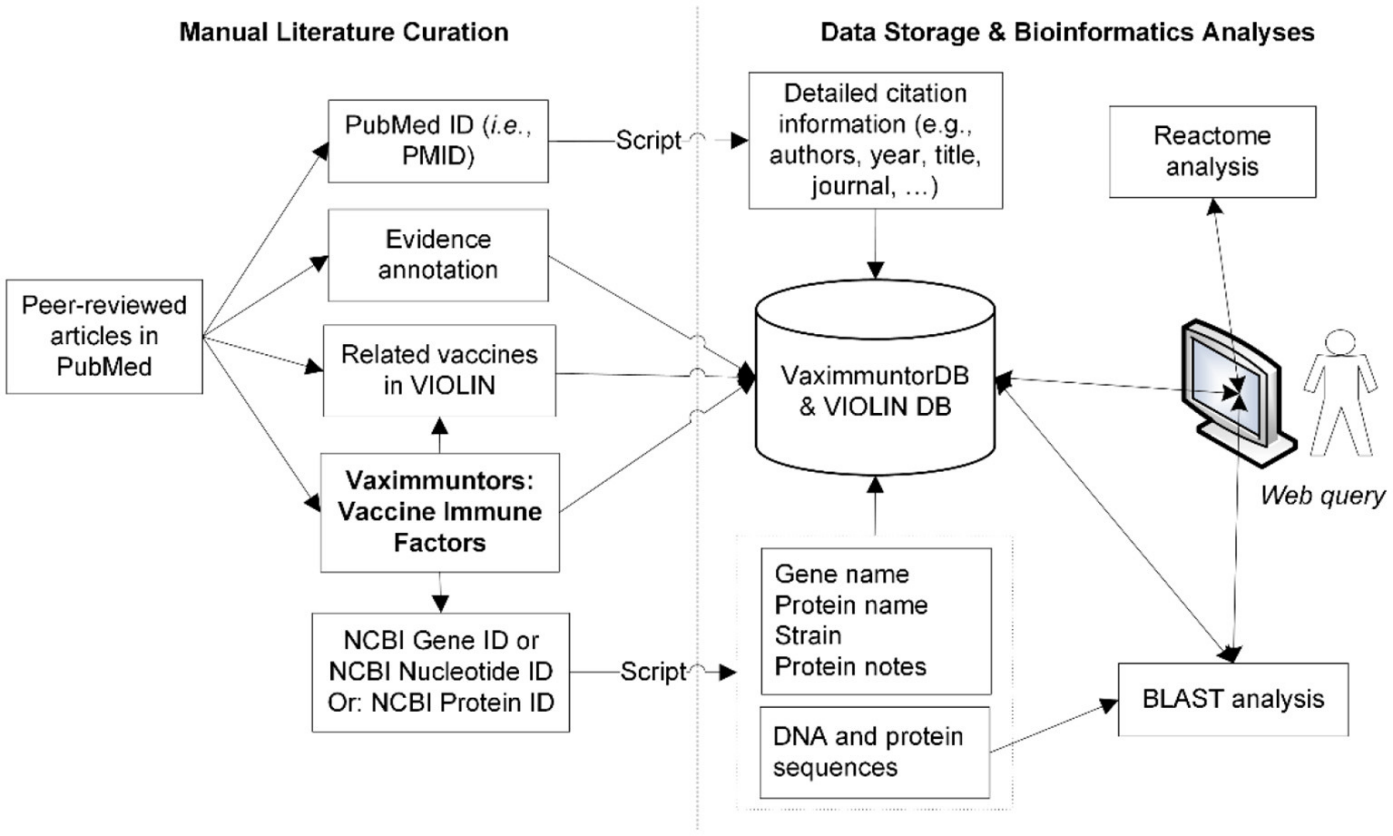
Overall system/architecture design. Vaximmutors are collected from manual curation of peer-reviewed articles available in PubMed. Gene names, NCBI Gene IDs, and gene fold changes were extracted and input into the VaximmutorDB. The NCBI Gene or Protein ID was used by a script that automatically extracts gene position, protein name, DNA sequence, and protein sequence from the NCBI Entrez Gene Database.

A semi-automatic data curation pipeline was implemented in VaximmutorDB (Figure 1). Specifically, a curator identified and manually annotated peer-reviewed articles from the PubMed literature database, and added to our internal web-based literature curation and analysis system (10, 11). In terms of immune responses, only PubMed results were annotated in our database since VaximmutorDB focuses on the annotation and collection of experimentally verified vaccine immune factors (vaximmutors) reported in original peer-reviewed journal articles. Meanwhile, the general vaccine information has been extracted from many other resources such as FDA package inserts. The gene and protein sequences were obtained from the NCBI Gene and Protein databases. Immune factors associated with all types of vaccines such as live or killed vaccines or subunit vaccines are all within our scope of annotation. The VaximmutorDB data annotation has started since 2010. Vaximmutor-related contents in the database include the vaccine name, vaximmutor gene name, Gene ID, PubMed PMID, vaccination route, host responses, and protection level. Vaximmutor-induced host responses are annotated as text description. With a PubMed PMID, our system is able to automatically retrieve all related citation information. With an identified Gene ID, our internal script could automatically retrieve gene name, protein name, species, DNA and protein sequences. A customized BLAST search was also developed. The system also allows an annotator to submit data and invite reviewers to check, edit, and approve the submitted annotations. Only after the review and approval of a domain expert, information will be integrated and made available in the VaximmutorDB website for outside users to query and browse.

### Gene Ontology (GO) Enrichment Analysis

Gene IDs for vaximmutors were extracted from the VaximmutorDB, and the GO enrichment analysis was conducted using the web-based DAVID bioinformatics service (12) and results were then downloaded. GO terms with False Discovery Rate (FDR) (13) adjusted *p*-value < 0.05 were submitted to the GOFox program for visualization (http://gofox.hegroup.org/).

### Reactome Pathway Analysis

Pathway analysis was conducted using Reactome (https://www.reactome.org) (6), the most comprehensive open source biological pathway knowledgebase. We used ReactomeFIViz (Version 8.0.0 with pathways released in December, 2019 from Reactome release 71), Rectome Cytoscape app, for pathway enrichment analysis after collecting vaximmutors for specific studies from the VaximmutorDB and mapping human Gene IDs to gene symbols (Supplementary File 1). The analysis results were explored from ReactomeFIViz for overlap analysis using R (https://www.r-project.org). For Fisher's exact test, we used 1,769 for the number of total human pathways (Reactome release 71, disease pathways were excluded) and 20,490 for human protein coding genes based on reviewed UniProt records collected in the Reactome database. FDR adjusted *p*-values were calculated and applied for pathway enrichment analysis results. Pathways having FDR values < 0.05 were selected as significant pathways.

### Two Use Case Studies

To showcase the utilities of VaximmutorDB, two specific use cases were studied. One use case was to compare and analyze the similarities and differences of the vaximmutors derived from two studies of clinical human responses to the same type of yellow fever vaccine (YF-Vax) (14, 15). Another use case is the comparative analysis of vaximmutors associated with live attenuated and killed inactivated influenza vaccines (16, 17).

## Results

### VaximmutorDB Database Statistics

VaximmutorDB currently stores 1,740 vaccine immune factors from 13 host species (e.g., human, mouse, and pig), annotated from over 200 peer-reviewed journal publications. The VaximmutorDB web query interface allows users to identify vaximmutors for specific hosts. For example, VaximmutorDB includes 1,691 human vaximmutors, 30 mouse vaximmutors, and 4 monkey vaximmutors.

The vaximmutors stored in VaximmutorDB are triggered by 154 various types of vaccines for 46 pathogens. These vaccines cover various vaccine types such as live attenuated vaccines, killed inactivated vaccines, recombinant protein vaccines, DNA vaccines, and recombinant vector vaccines. The database architecture, and the contents and flows of our manually annotated data are illustrated in Figure 1.

### Top Vaximmutors Showing Most Influential Vaccine Immune Factors

Table 1 lists the top 10 vaximmutors identified to be associated with most vaccines in VaximmutorDB (10, 11). Among the top 10 vaximmutors are three antibodies (IgG, IgG2a and IgG1), two T helper type 1 (Th1) cytokines (IFN-γ and IL-2) (18), two T helper type 2 (Th2) cytokines (IL-4 and IL-6) (18), and three innate immune response factors (TNF-α, CASP-1, and TLR8). All these vaximmutors are up-regulated after some vaccination stimulation, except that TNF-α, IL-4, and TLR8 are sometimes down-regulated as well (Table 1). Note that our up- or down-regulated vaximmutor expression results only include those triggered by direct vaccination, and they do not include the responses stimulated by vaccination followed by virulent pathogen challenge. The up- or down-regulation of these vaximmutors indicate different types of vaccine immune responses as briefly introduced below.

**Table 1:** Top 10 vaximmutors defined by number of associated vaccines.

**Category**	**Vaximmutor**	**Host species**	**Associated vaccines**	**NCBI gene ID**	**Description**
Antibodies	IgG	Mouse	37 (all up)	16059	Antibody
	IgG2a	Mouse	18 (all up)	668478	Antibody
	IgG1	Mouse	15 (all up)	16017	Antibody
Th1 cytokines	IFN-gamma	Mouse	47 (all up)	15978	Pro-inflammatory Th1 response
	IL-2	Mouse	8 (all up)	16183	Th1, T cell stimulation
Th2 cytokines	*IL-4*	Mouse	12 (8 up, 4 down)	16189	Th2, IgE production
More innate immune response factors	IL-6	Mouse	8 (all up)	16193	Th1/Th2 differentiation
	TNFα	Mouse	11 (7 up, 4 down)	21926	Pro-inflammatory Th1 response
	CASP-1	Human	5 (all up)	834	Pro-inflammatory, pyroptosis
	TLR8	Human	5 (4 up, 1 up and down at different days)	51311	Adjuvant effect, NF-KB pathway activation, PAMP recognition

Humoral antibody response is a critical vaccine-induced response. The 2nd−4th most common vaximmutors are IgG, IgG2a, and IgG1, which are antibodies produced by B cells. IgG antibodies have a high specific affinity for pathogen antigens. IgG antibodies are also involved in the secondary immune response and help to establish immunological memory. The presence of these antibodies highlights the significance of the antibodies in establishing a long-term immunological memory to fight infection. IgG2a and IgG1 are produced by antigen-specific B cells induced by Th1 and Th2 cells, respectively (19). As a result, IgG2a and IgG1 have been frequently used to test Th1 and Th2 responses (20, 21).

Th1 and Th2 immune responses have been critical to vaccine responses. Th1 responses stimulate cell-mediated cytotoxic T lymphocyte (CTL) activity, and Th2 responses are critical to stimulate antibody responses. The induction of the Th1 response by vaccines is therefore important to the clearance of the corresponding intracellular pathogens from the host. Interferon gamma (IFN-γ), the most common vaximmutor found in our database, is a cytokine of the Th1 immune response, which promotes activation and production of macrophages and natural killer cells. IFN-γ enhances the production of IgG2a. As a pro-inflammatory cytokine, IFN-γ helps to destroy pathogens by increasing phagocytosis, forming granulomas, and promoting MHC II expression (18). IFN-γ also synergistically works with TNF-α (the fifth most common vaximmutor) to promote the Th1 response against pathogen infection. The roles of TNF-α also include maintaining granulomas, an important defense mechanism against mycobacteria (22). Interleukine-4 (IL-4), the 6th most common vaximmutor, is an important cytokine involved in activating Th2 cells. IL-4 promote B cell response and production of IgG1 and IgE antibodies (18, 19). IL-4 can also be inhibitory to the Th1 response (23). IL-6 has multiple roles. It may function as a pro-inflammatory or an anti-inflammatory cytokine. IL-6 participates in acute phase, response, macrophage differentiation, and B cell maturation. IL-6 also controls the Th1/Th2 differentiation by promoting Th2 differentiation and inhibiting Th1 polarization (24).

CASP-1 and TLR8, the two other innate immune response factors identified in our database, can also stimulate strong pro-inflammatory responses and are important to fight against various diseases (25, 26). Like IFN-γ and TNF-α, Caspase-1 (CASP-1) also promotes proinflammatory responses. CASP-1 induces the secretion of the proinflammatory cytokines IL-1β and IL-18, and pyroptosis, a form of proinflammatory cell death induced by bacterial pathogens (27–30). The frequent finding of CASP-1 in VaximmutorDB suggests the importance of CASP-1 in protective vaccine immunity. Toll-like receptor 8 (TLR8) agonists are potent vaccine adjuvants, and the TLR8 bound human agonists induce the maturation of human myeloid dendritic cells (mDCs) and plasmacytoid DCs (pDCs) that link innate and adaptive immunity (31). Extending the traditional Th1 and Th2 cell dichotomy, the third distinct lineage of CD4 T helper cells called T helper 17 (Th17) cells have been discovered (32–34). Th17 cells produce effector molecules IL-17, IL-17F, IL-21, and IL-22. Th17 cells and their induced cytokines have been found critical for vaccine-induced memory immunity against a variety of pathogens, primarily at mucosal sites (35). VaximmutorDB includes IL-17 (36–38) and IL-17F (39) as vaximmutors; however, they are not ranked as high based on their associated vaccines.

### Enriched Vaccine Immune Responses Analyzed Using All Vaximmutors

**Figure 2:**
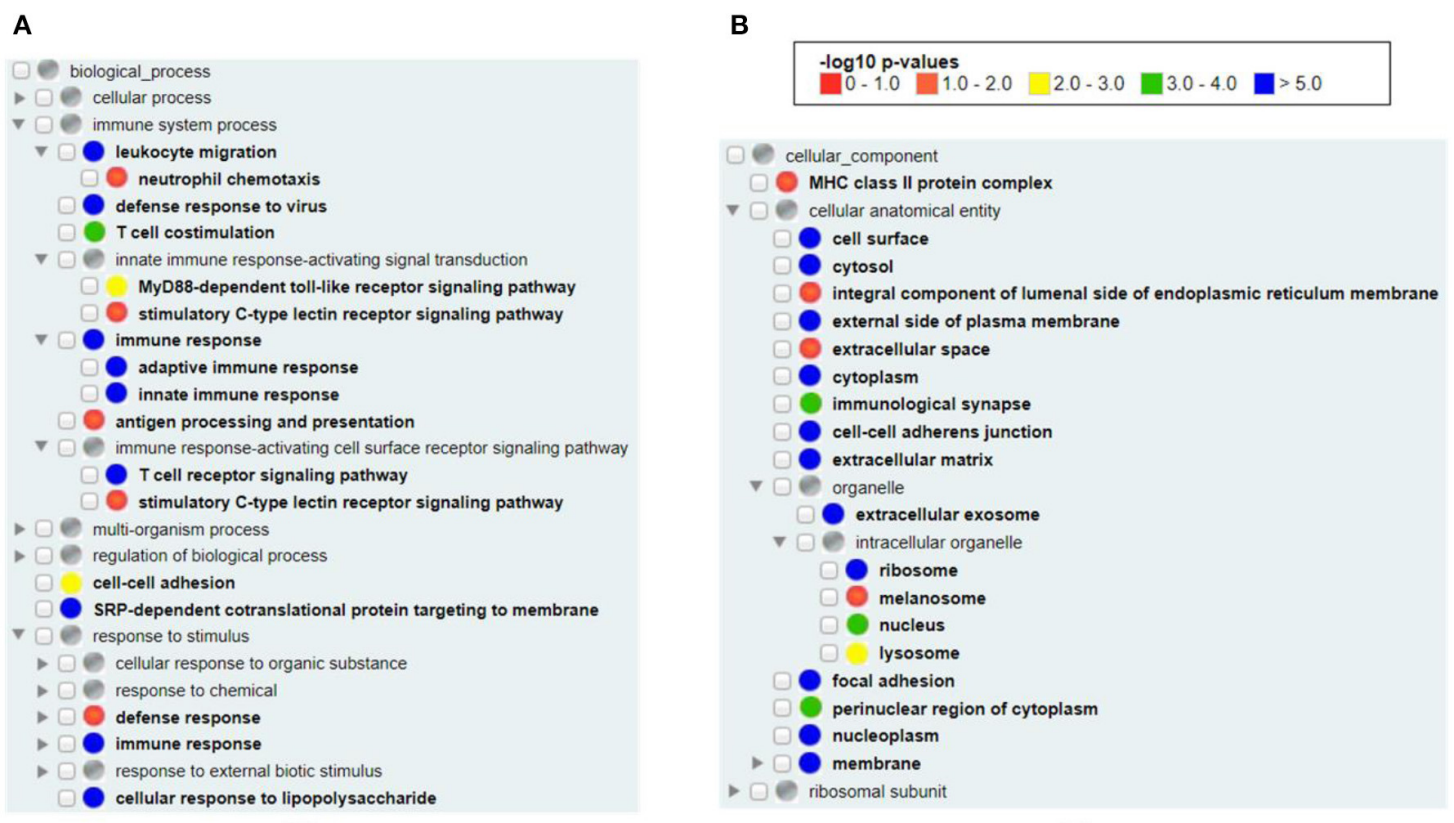
GO enrichment analysis of all human vaximmutors. All available 1,675 human vaximmutors were extracted and analyzed using DAVID tool, and the enriched GO biological processes **(A)** and cellular components **(B)** were processed and visualized using GOfox. The *p*-value bar represented the color representation scheme of different adjusted *p*-value ranges. The cutoff was set as 0.05.

Our Gene Ontology (GO) enrichment analysis of all available human vaximmutors identified the most enriched GO biological process and cellular component terms (Figure 2). As expected, both the innate and adaptive immune responses (both *p*-value < 10^−5^) as well as antigen processing and presenting (adjusted *p*-value < 0.05) were enriched (Figure 2A). MyD88-dependent Toll-like receptor (*p*-value < 0.001) and stimulatory C-type lectin receptor (*p*-value < 0.05) signal pathways are identified to be significantly enriched. Cellular response to lipopolysaccharide is identified, suggesting the critical role of bacterial lipopolysaccharide to vaccine-induced immunity. It is unexpected to find the process of “SRP-dependent cotranslational protein targeting to membrane” in our analysis (*p*-value < 10^−5^). The signal recognition particle (SRP) and its membrane-bound receptor (SR) deliver membrane proteins and secretory proteins to the endoplasmic reticulum (ER) membrane (40). The SRP pathway is a main mechanism that targets polypeptides in Gram-positive bacteria. In *Streptococcus pyogenes*, the SRP pathway is required for the secretion of a subset of virulence factors, the *in vivo* survival, and the pathogenesis of bacterium (41). Peptides loading on the MHC class I molecule occurs in the ER, suggesting that the transportation of antigens to ER is critical (42). It is possible that vaccine antigens undergo both the SRP pathway and antigen presentation pathway and there is an intricate interaction between the two pathways. In cellular components, we found the enriched MHC class II (*p*-value < 0.05, but not class I) protein complex (Figure 2B), suggesting that MHC class II protein complex is more frequently found than MHC class I protein complex in general vaccine immune responses. MHC class II molecules are normally found on professional antigen-presenting cells such as dendritic cells and macrophages, and are important in initiating immune responses. Many organelles, including melanosome, lysosome, extracellular exosome, and nucleoplasm were also enriched in our analysis (Figure 2B).

### Pathway-Based Analysis of Vaximmutors Uncovering Molecular Mechanisms of Vaccine Response

**Figure 3:**
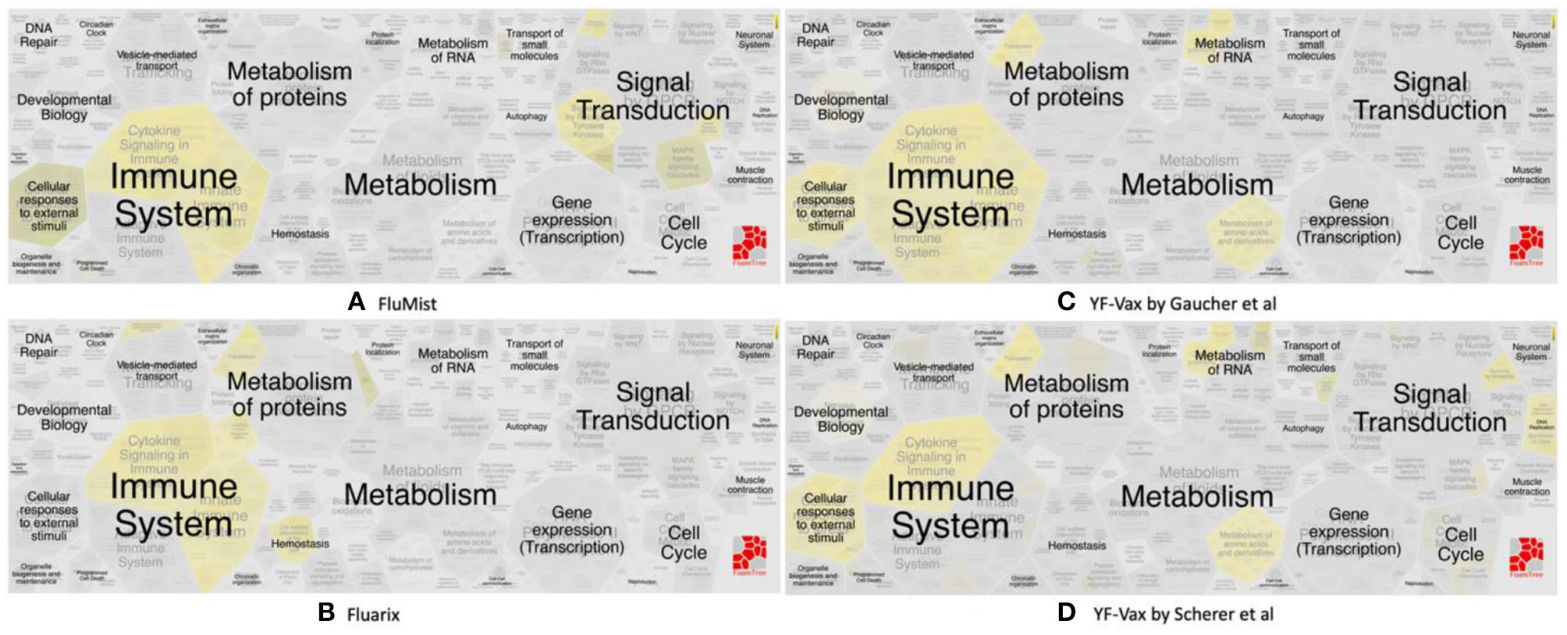
Reactome pathway enrichment analysis highlighting significant pathways of **(A)** FluMist (145 genes), **(B)** Fluarix (84 genes), **(C)** Gaucher et al. (15) (620 genes) and **(D)** Scherer et al. (14) (571 genes). The complexity of biomolecular pathways is organized by hierarchical groupings of the FoamTree (https://carrotsearch.com/foamtree/) and the color gradient highlights most to least significant pathways (*p*-value < 0.05).

The vaximmutors induced by influenza and yellow fever vaccines were extracted from the VaximmutorDB and specifically analyzed. By comparing their associated pathways, overlapping but different profiles were identified (Figure 3). These use cases are detailed below.

#### Use Case 1. Differential Immune Response Profiles of Vaximmutors Associated With Live Attenuated and Killed Inactivated Influenza Vaccines

Vaximmutors associated with influenza vaccines include 145 human genes induced by FluMist, a live attenuated influenza vaccine (LAIV) (17), and 84 human genes induced by Fluarix (16), a trivalent inactivated vaccine (TIV). These immune factors correlate with protection and have been compiled in this study. It is interesting that our previous ontology-based study found that LAIV is safer than TIV in terms of their association with severe adverse events (43). Note that the LAIV vaccination is not recommended for children and adults over the age of 49 years. Therefore, more comprehensive studies and comparisons are needed to compare the safety profiles between LAIV and TIV under specific conditions.

Significant similarities and differences between FluMist and Fluarix-induced vaximmutors pathways were identified (Figures 3A,B, Supplementary File 2). Both FluMist and Fluarix stimulated the signaling pathways of many interleukins (including IL-1, IL-4, IL-6, IL-13, IL-20, and IL-27), interferon signaling, MARK1 activation, neutrophil degranulation, and innate immunity (Supplementary File 2). These shared enriched immune responses appear to be crucial to the protection against influenza infection.

Many unique profiles were stimulated by live attenuated vaccine FluMist and killed inactivated vaccine Fluarix. Overall FluMist stimulated a much wider response than Fluarix (Figures 3A,B and Supplementary Figures 1, 2). Particularly, FluMist stimulated statistically significantly enriched responses in the areas of cytokine signaling in immune system, signaling by receptor tyrosine kinases, death receptor signaling, and cellular responses to external stimuli (Figure 3A). There are 10 Toll like receptors (TLRs) in humans that recognize various structurally conserved molecules from microbes. Interestingly, FluMist stimulated all types of signaling cascade pathways of TLRs except TLR1 with much lower *p*-values than Fluarix (Supplementary File 2). NOD1 and NOD2, two intracellular pattern recognition receptors, were also significantly induced by FluMist. FluMist also stimulated many important immune cytokines including TNF, and Interferon alpha/beta. In contrast, Fluarix stimulates many pathways such as those involved in protein metabolism, vesicle-mediated transport, and hemostatsis (Figure 3B and Supplementary Figure 2). The immune responses stimulated by Fluarix focus on complement cascade and its regulation, and B cell receptor signaling (Supplementary File 2).

#### Use Case 2. Common Vaccine Response Mechanisms Revealed by Pathway-Based Comparison Analysis of Two YF-Vax Vaccine Response Studies

Live attenuated yellow fever virus vaccine YF-17D and its substrains (including YF-Vax) derived from the original 17D strain (44) have been widely used for vaccination, and the immune responses in vaccinated humans tend to be strong (45, 46). YF-17D is an ideal vaccine model for protective immune mechanism studies (45, 46). To uncover the molecular mechanisms underlying YF-17D responses, we conducted pathway analysis and compared significantly enriched pathways using vaximmutors collected in VaximmutorDB from two studies: Scherer et al. (14) and Gaucher et al. (15).

**Figure 4:**
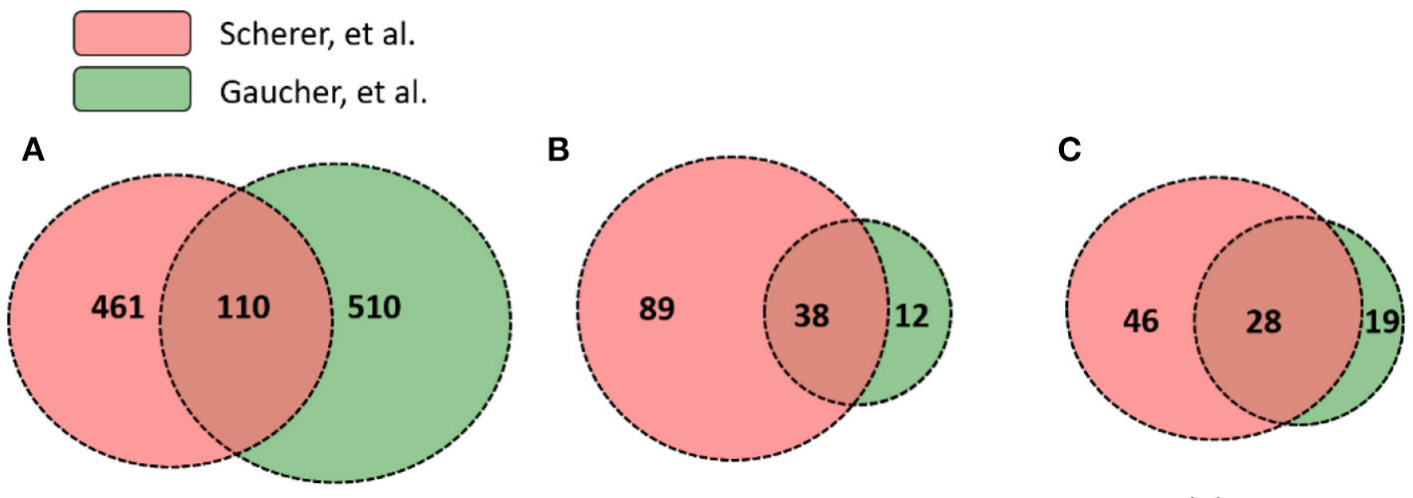
Gene and pathway level comparison between Scherer et al. (14) and Gaucher et al. (15). Both studies analyzed YF-Vax-induced immune responses in vaccinated human subjects. The comparison between these two studies based on: **(A)** numbers of significant genes; **(B)** enriched Reactome pathways based on both unique and shared genes; and **(C)** enriched Reactome pathways based on only unique genes (Note that 110 genes in A were not included for this analysis). Even when no shared genes are analyzed, many pathways are shared as seen in **(C)**.

While significantly expressed genes from two studies differed, their pathway results appeared to be more conserved than the gene list difference. Scherer et al. (14) and Gaucher et al. (15) identified 571 and 620 differentially expressed genes in human subjects' immune response that were vaccinated with the same yellow fever vaccine YF-Vax, respectively (Supplementary File 1). Among these genes, 110 were shared, 461 unique in Scherer et al. (14), and 510 unique in Gaucher et al. (15) (Figure 4). Though the two sets of vaximmutors are significantly overlapped (*p*-value < 2.2E-16), the number of shared vaximmutors is low: 19% Scherer et al. (14) study and 18% for Gaucher et al. (15) studies (Figure 4A). Meanwhile, we performed pathway enrichment analysis and compared the significant pathways using Reactome's ReactomeFIViz app. Our results identified more shared pathways than shared genes (Figure 4, Supplementary File 3). Our results (Supplementary File 3) uncover 127 significant pathways for Scherer et al. study and 50 significant pathways for Gaucher et al. (15) study. Among these pathways, 38 are shared between these two studies (*p*-value < 2.2E-16) (Supplementary File 3). Compared to gene-based overlap analysis results, pathway-based analysis results show much higher shared ratios: 30 and 76% for Scherer et al. study and Gaucher et al. (15) study, respectively. The similar pathway profiles shown up in Figures 3C,D (Supplementary Figure 3) also confirmed the pattern.

To further provide evidence showing that pathway-based analysis is better to show common vaccine response mechanisms than using genes alone, we conducted another pathway enrichment and comparison analysis using vaximmutors that are not shared in these two studies. Reactome analysis tool produced 74 significant pathways for Scherer et al. study and 47 for Gaucher et al. (15) study (Figure 4C, Supplementary File 4). Among these pathways, 28 are shared (*p*-value < 2.2E-16), the majority of which are related to metabolisms of proteins and RNAs, implying a higher activity of synthesis of proteins during vaccine response. Interestingly all these 28 shared pathways (Figure 4C) are included in 38 shared pathways (Figure 4B) produced using all vaximmutors without removing shared ones, implying a significant portion of useful information are provided from these vaximmutors not shared by these two studies.

**Figure 5:**
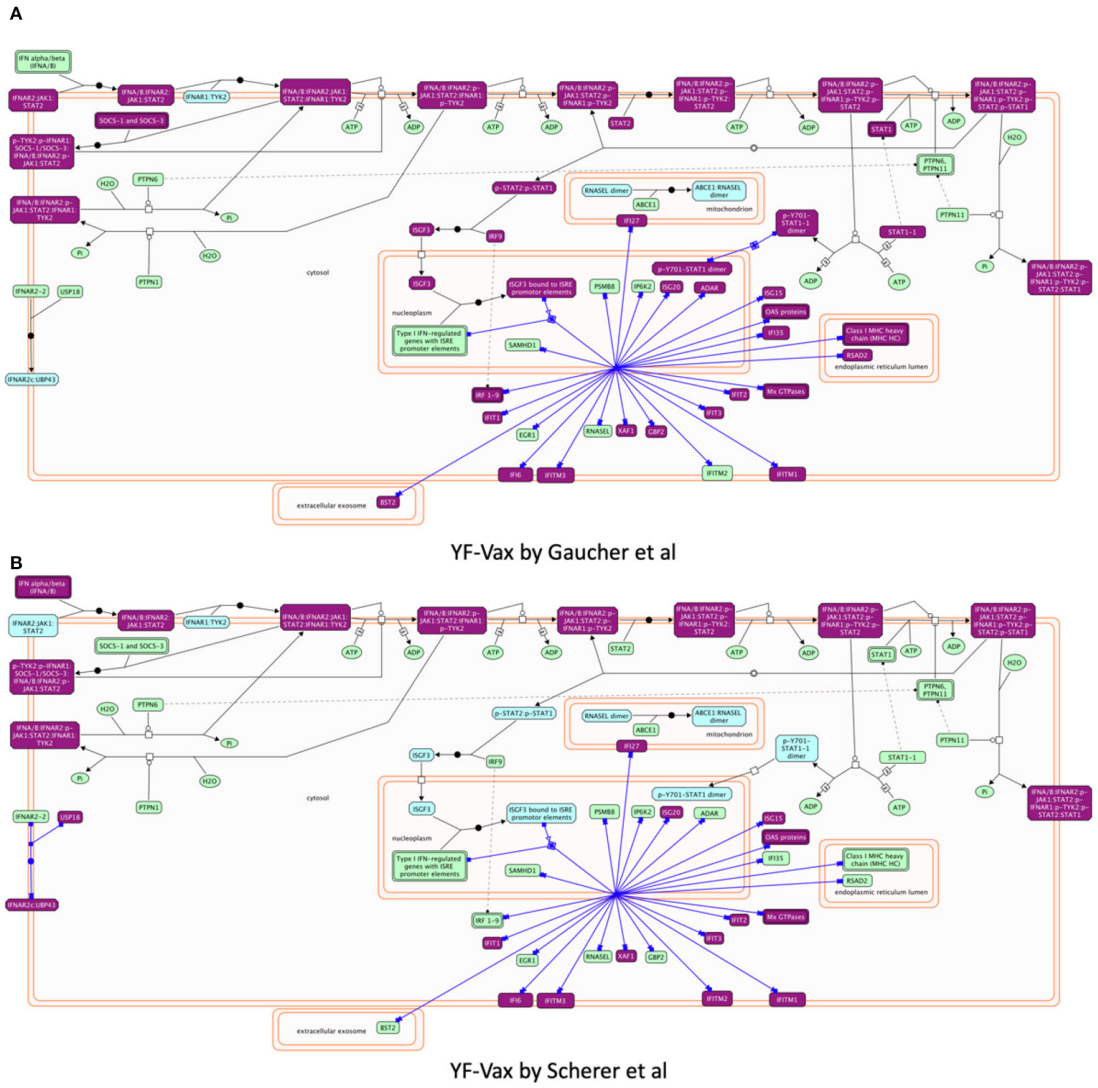
Reactome pathway enrichment analysis highlighting Interferon alpha/beta Signaling pathways found to be significant in both **(A)** Gaucher et al. (15) and **(B)** Scherer et al. (14). The pathway diagram is in the Systems Biology Graphical Notation (SBGN) (47) format. Results from both studies imply the stimulation of expression of IFN-induced genes though the Gaucher et al. (15) study shows more hit genes than the Scherer et al. study. Intriguingly, the Gaucher et al. (15) study implies STAT1, which is not shown in the Scherer et al. (14) study, while the Scherer study has USP18 that is not shared in the Gaucher et al. (15) study.

Reactome provides biochemical reactions-based pathway diagrams based on the SBGN standard (47) to help users understand enrichment analysis results. For example, focusing on pathways annotated under Interferon Signaling, a sub-pathway of Cytokine Signaling in Immune System and a significant pathway for both studies, we found IFN α/β signaling showing greater involvement in Gaucher et al. (15) (Hits: 27/69, FDR = 4.33E-15) than in Scherer et al. (14) (Hits: 20/69, *p*-value = 3.10E-11) though it is a significant pathway for both studies. Investigating the detailed biochemical reaction-based pathway diagram for Interferon Alpha/Beta Signaling (Figures 5A,B), we found that most of the vaximmutors from both studies are related to a reaction called “Expression of IFN-induced genes” (https://reactome.org/PathwayBrowser/#/R-HSA-909733&SEL=R-HSA-1015702), presumably the reason why this pathway is a significantly enriched pathway for both studies, though the Gaucher et al. (15) study has more hits than the Scherer et al. (14) study. However, intriguingly, the Scherer et al. (14) study has also implied the involvement of USP18, which is not shown in the Gaucher et al. (15) study, while the Gaucher et al. (15) study has STAT1 that is not shown in the Scherer et al. study.

### VaximmutorDB Web Query and Data Integration

**Figure 6:**
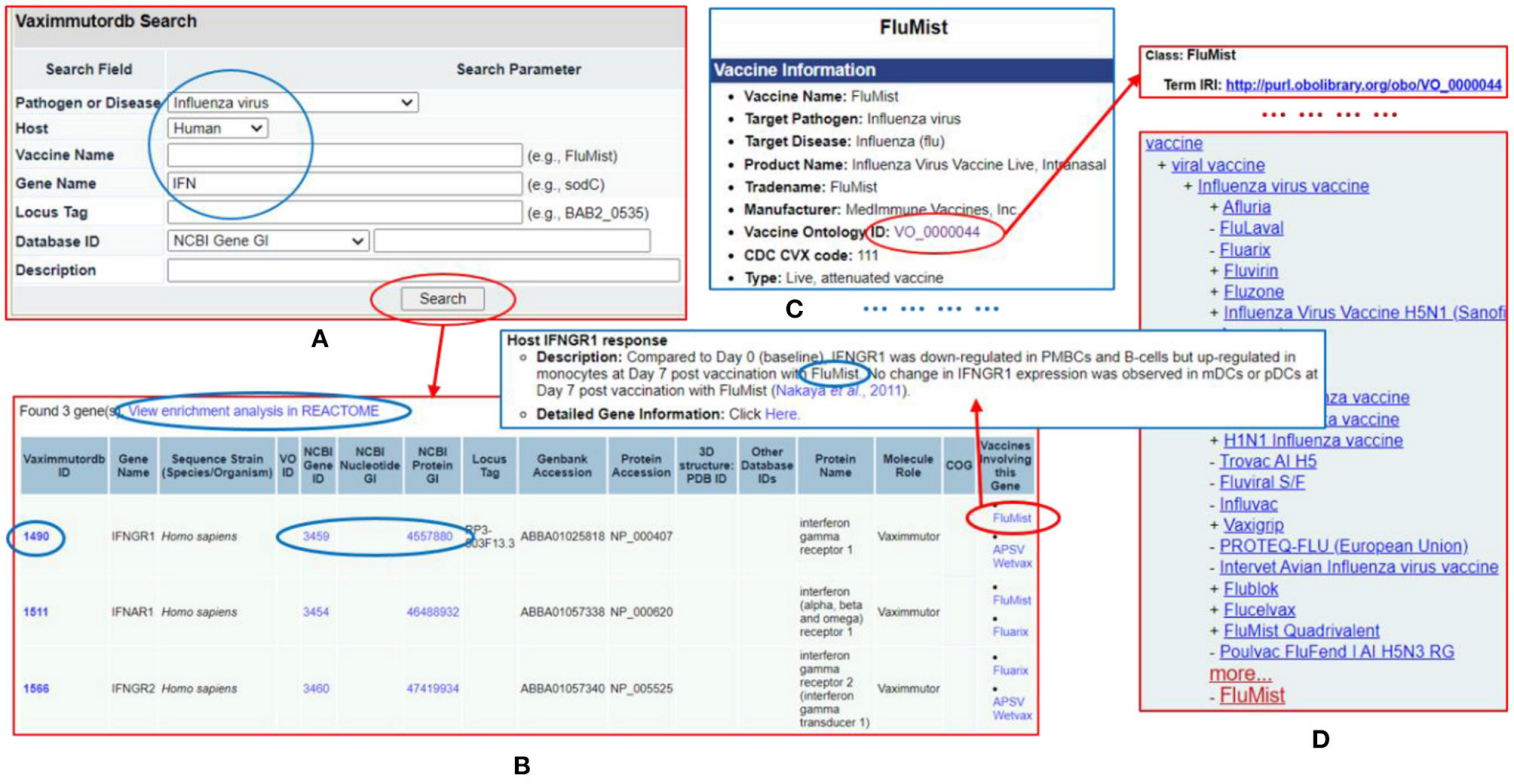
VaximmutorDB web query example. **(A)** Search VaximmutorDB for influenza virus (pathogen), human (host), and gene names containing “IFN.” **(B)** Three genes are found and each gene is associated with two influenza vaccines. **(C)** The detailed vaccine information and how the gene was regulated in vaccinated hosts are provided in another VaximmutorDB page. **(D)** A click of the VO ID leads to an Ontobee Ong et al. (48) web page showing the information about this vaccine. There are many links on different pages to useful information.

The Vaximmutor data collected in VaximmutorDB are freely available for download via the website as described in the Methods section. The manually-curated and pre-computed VaximmutorDB data can be efficiently queried and visualized (Figure 6). Specifically, the VaximmutorDB web query system allows querying of various vaximmutors, related vaccines, gene and protein details, and manually annotated host vaximmutor response information (Figures 6A,B). Whenever possible, the website also provides the Vaccine Ontology (VO) IDs and links to the detailed ontology pages (Figures 6C,D). The users can also perform pathway-enrichment analysis using the Reactome's analysis tool directly from this web site.

## Discussion

The contribution of this paper is multiple. First, we for the first time introduce VaximmutorDB, a novel web-based database focusing on the manual annotation and analysis of vaccine immune factors named as vaximmutors. To our knowledge, VaximmutorDB is the first web-based database of such vaccine immune factors. A total of over 1,700 vaximmutors were collected and manually annotated. Second, our systematic analysis of vaximmutors identified many insights in terms of the mechanisms of vaccine-induced responses. Top 10 vaximmutors and GO enriched patterns with all the vaximmutors were identified and their contributions to vaccine protection analyzed. The different vaximmutor responses by two different types (live attenuated and killed inactivated) of influenza vaccines and one type yellow fever vaccine (i.e., YF-17D) but in different studies were also systematically analyzed and reported. The database query and data sharing format are also provided.

Specific patterns have been identified from our analyses of all the collected Vaximmutors. For example, our top 10 vaximmutors identified IgG, IgG2a, and IgG1, and many Th1 and Th2 cytokines, which are critical to protective vaccine immune responses (Table 1). Pro-inflammatory factors, such as CASP-1, TNFα, and IFN-γ, are among the top 10 vaximmutors. Our GO enrichment analysis identified many enriched biological processes and cellular components (Figure 2). In addition to generally known processes such as innate and adaptive immune responses, many uncommon processes such as “stimulatory C-type lectin receptor signaling pathway” and “SRP-dependent cotranslational protein targeting to membrane” are also found. C-type lectin receptors expressed by dendritic cells are critical to regulate the induction of signaling pathways that further regulate adaptive immune responses (4). Signal-recognition particle (SRP) is a cytosolic particle that transiently binds to the ER signal sequence in a nascent protein. SRP-dependent cotranslational proteins targeting to membrane appear to be important to viral immune responses (49). How these pathways are related to vaccine protection deserves further investigation.

Vaccine-induced immune responses appear to differ given different types of vaccines (Figure 3). Live attenuated (FluMist) and killed inactivated (Fluarix) appeared to induce overlapping host responses. While they shared enriched immune system responses, FluMist induced significant signal transduction responses (Figure 3A), and Fluarix induced significant metabolism of protein responses (Figure 3B). These results suggested that the live attenuated influenza vaccine underwent many signal transduction processes after vaccinated, and the killed inactivated vaccine stimulated metabolic processes to digest the killed viruses. The signal transduction from LAIV and metabolic process from TIV might significantly impact the vaccine safety and efficacy. The two Yellow Fever vaccine studies appeared to show very similar vaccine-induced responses in the vaccines (Figures 3C,D). Strong immune system responses were stimulated. Different from the live attenuated influenza vaccine FluMist, the Yellow Fever live attenuated vaccine stimulated strong metabolism responses similar to the killed vaccine Fluarix. Interestingly, the Yellow Fever vaccine and Fluarix both stimulated “SRP-dependent cotranslational protein targeting to membrane” (*p*-value < 0.001) (Supplementary Files 2, 3), which were consistent with the observation found with all the vaximmutors (see above). Therefore, the vaccine immune patterns can be quite different depending on the vaccine and pathogen types. Note that some specific responses, such as interferon signaling, appeared to be shared by all influenza and Yellow Fever vaccines. More detailed studies on the similarities and differences are required to understand the underlying causal mechanisms.

More exploration is needed to understand the interesting observation that killed inactivated Fluarix (Figure 3B) and live attenuated YF-Vax (Figures 3C,D), but not live attenuated FluMist (Figure 3A), simulate significant responses in the metabolism of proteins. The results suggest that killed inactivated and live attenuated vaccine antigens are able to induce similar responses such as the metabolism responses, which is likely significantly influenced by their associated vaccine adjuvants and immune stimulators. In addition, the route of vaccination may play a major role. YF-Vax is administered through subcutaneous rote, and Fluarix through intramuscular injection. While the live/killed phenotype and pathogen type of vaccines differ, the route of vaccination causes more confusion for the final outcome analysis.

Our Yellow Fever vaccine study showed that the administration of the same vaccine can result in different outcomes as identified in the two different high throughput studies. In our previous study (50), we applied the community-based Ontology of Biological and Clinical Statistics (OBCS) (50, 51) and Vaccine Ontology (52, 53) to study how different variables could change the identification of different vaccine immune factors in three yellow fever vaccine studies (14, 15, 54). Our ontology-based meta-analysis of the variables identified over 20 variables whose values differed across these three different studies (50). Examples of these variables include different human subjects, sample preparation, RNA preparation, microarray chip type, raw data process software, and analysis settings. The differences in experimental conditions might have caused the different vaccine immune response studies.

Meanwhile, our current study found that although the gene lists differ greatly from two different studies, the enriched pathways from these studies showed greater similarities (Figure 4). It appears that although different genes were up- or down-regulated given different conditions, these genes participated in the similar set of pathways. In addition to the usage of the manually annotated VaximmutorDB knowledge, a previous direct analysis of raw OMICS data from different studies also resulted in the same conclusion (i.e., more shared pathways than shared gene lists) (55). Therefore, it is important to map genes to pathways and conduct pathway-based analysis and visualization of vaximmutors. Reactome is an important source for pathway analysis. Our results indicate that vaximmutors collected in VaximmutorDB combined with Reactome's multi-scale pathway perspectives facilitate functional analysis, allowing researchers to elucidate overarching molecular mechanisms within shared pathways that would not be possible by looking at vaximmutors alone.

More vaximmutor-related research is needed to further understand the vaccine-induced immune mechanisms. Since we now know the different vaccine immune results given different experimental factors, we may be able to find more about the close relations among vaccines, vaximmutors, and pathways. It is important to identify specific vaximmutor gene markers that correlate with protection and identify vaccine-specific pathway patterns. More data, such as the high throughput data from the ImmPort immunology database and analysis portal (https://ImmPort.niaid.nih.gov/) (56, 57), can be systematically analyzed for such investigations. Ontologies such as OBCS, VO, Vaccine Investigation Ontology (VIO) (55), and Cell Ontology (58) can be used to standardize the data for efficient secondary data analyses. The gene expression results can be from transcriptomical or proteomic analysis. It would be ideal to differentiate different analyses, and ontology can support the standard representation and classification. The ontology-based high throughput gene expression data annotation and analyses are expected to further enhance our understanding of the vaccine immune mechanisms.

The VaximmutorDB database will undergo continuous extension and analysis. We will keep adding more vaximmutors and associate them with more vaccines and associated information. We are also in the process of linking and integrating various vaximmutors to existing databases such as ImmPort portal and Reactome pathway database. The VaximmutorDB will include any relevant data on newer vaccines aimed at targeting COVID-19. It will analyze the immune responses elicited by the new COVID vaccines and compare them to the immune response of past vaccines.

In conclusion, VaximmutorDB is the first web-based database of vaccine immune factors named vaximmutors. A total of over 1,700 vaximmutors from 13 host species (e.g., human, mouse, and pig) were collected, manually annotated, and further analyzed using bioinformatics approaches. Top 10 vaximmutors and the enriched patterns with all the vaximmutors were identified. Overlapping and differential gene lists and enriched pathways induced by live attenuated and killed inactivated influenza vaccines and yellow fever vaccines were identified and systematically analyzed. User-friendly database query and standard data sharing formats are provided. VaximmutorDB provides a powerful platform for annotation, standardization, storage, and analysis of vaccine immune factors, leading to the better understanding of the fundamental protective vaccine immunity.

## Data Availability Statement

The original contributions presented in the study are included in the article/supplementary material, further inquiries can be directed to the corresponding author/s.

## Author Contributions

KB, PS, JO, and RR collected and annotated data. EO, NS, AH, TB, and FL performed bioinformatics data. EO, BZ, and ZX generated the website. AM, JZ, and YH provide domain expert data interpretation. GW and YH contributed to the study design. KB, EO, NS, GW, and YH wrote the manuscript. All authors performed result interpretation, discussed, and reviewed the manuscript.

## Conflict of Interest

The authors declare that the research was conducted in the absence of any commercial or financial relationships that could be construed as a potential conflict of interest.

## Supplementary Material

Supplementary Figure 1 More detailed results of the Reactome pathway enrichment analysis highlighting significant pathways of FluMist (145 genes). The complexity of biomolecular pathways is organized by hierarchical groupings of the FoamTree (https://carrotsearch.com/foamtree/) and the color gradient highlights most to least significant pathways (*p*-value < 0.05).

Supplementary Figure 2 More detailed results of the Reactome pathway enrichment analysis highlighting significant pathways of Fluarix (84 genes). The complexity of biomolecular pathways is organized by hierarchical groupings of the FoamTree (https://carrotsearch.com/foamtree/) and the color gradient highlights most to least significant pathways (*p*-value < 0.05).

Supplementary Figure 3 More detailed results of the Reactome pathway enrichment analysis highlighting significant pathways induced by YF-Vax-induced immune factors as identified in Gaucher et al. (15) (620 genes). The complexity of biomolecular pathways is organized by hierarchical groupings of the FoamTree (https://carrotsearch.com/foamtree/) and the color gradient highlights most to least significant pathways (*p*-value < 0.05).

Supplementary Figure 4 More detailed results of the Reactome pathway enrichment analysis highlighting significant pathways induced by YF-Vax-induced immune factors as identified in Scherer et al. (14) (571 genes). The complexity of biomolecular pathways is organized by hierarchical groupings of the FoamTree (https://carrotsearch.com/foamtree/) and the color gradient highlights most to least significant pathways (*p*-value < 0.05).

Supplementary File 1 Specific gene lists identified for four vaccine immune factor studies: FluMist (145 genes), FluMist (145 genes), Gaucher et al. (15) (620 genes), and Scherer et al. (14) (571 genes).

Supplementary File 2 Significantly regulated Reactome pathways enriched by Flumist-induced vaximmutors, Fluarix-induced vaximmutors, and the shared pathways by both Flumist- and Fluraix-induced vaximmutors.

Supplementary File 3 Significantly regulated Reactome pathways enriched by vaximmutors found in Gaucher et al. (15) (620 genes), Scherer et al. (14) (571 genes), and shared by both studies.

Supplementary File 4 Significantly regulated Reactome pathways enriched by vaximmutors found vaximmutors that are not shared in these two studies Gaucher et al. (15) and Scherer et al. (14).
