# Supplementary figures and images for: VaximmutorDB: A Web-Based Vaccine Immune Factor Database and Its Application for Understanding Vaccine-Induced Immune Mechanisms
Source: Front Immunol. 2021 Mar 12;12:639491. doi: 10.3389/fimmu.2021.639491 (PMC7994782; doi:10.3389/fimmu.2021.639491)

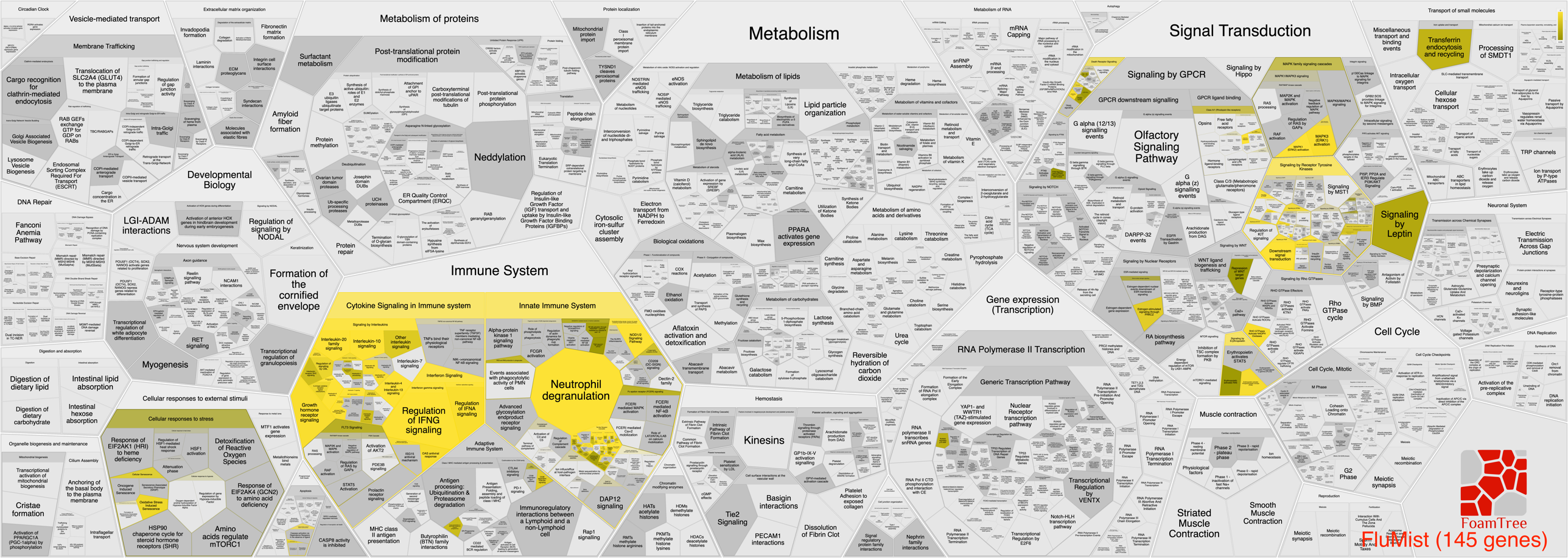

Supplement: Supplementary Figure 1 — More detailed results of the Reactome pathway enrichment analysis highlighting significant pathways of FluMist (145 genes). The complexity of biomolecular pathways is organized by hierarchical groupings of the FoamTree (https://carrotsearch.com/foamtree/) and the color gradient highlights most to least significant pathways (p-value < 0.05). [file Image_1.PNG]

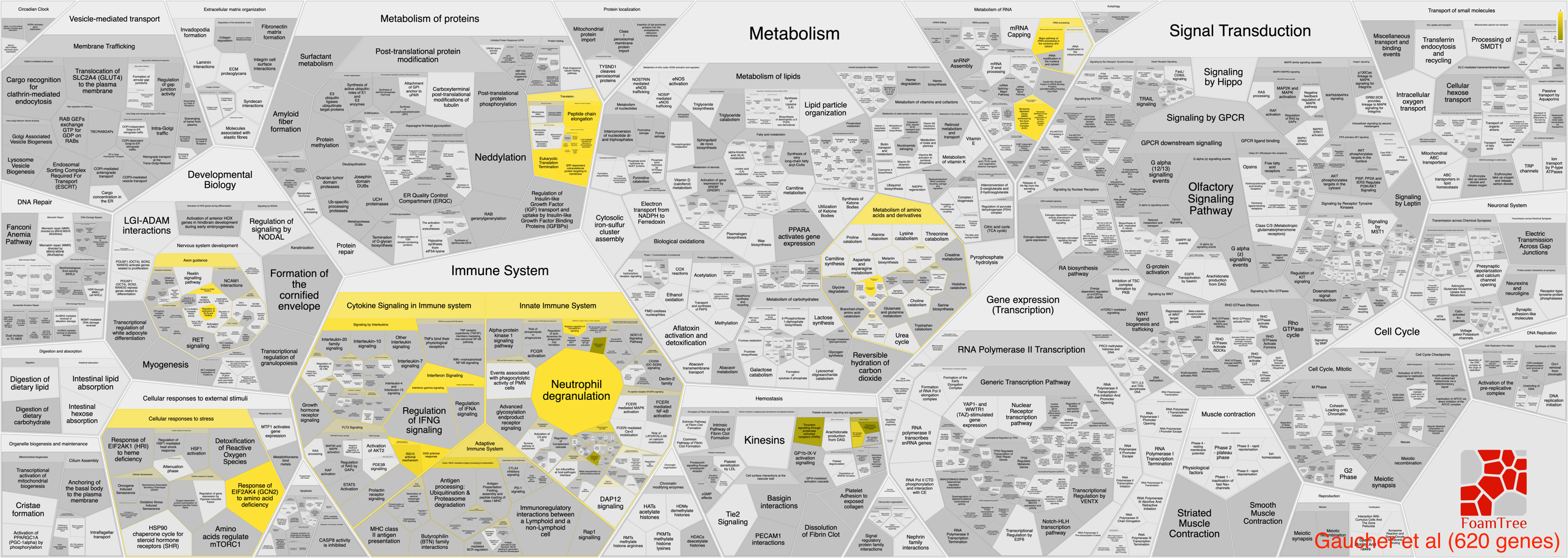

Supplement: Supplementary Figure 3 — More detailed results of the Reactome pathway enrichment analysis highlighting significant pathways induced by YF-Vax-induced immune factors as identified in Gaucher et al. (15) (620 genes). The complexity of biomolecular pathways is organized by hierarchical groupings of the FoamTree (https://carrotsearch.com/foamtree/) and the color gradient highlights most to least significant pathways (p-value < 0.05). [file Image_3.PNG]

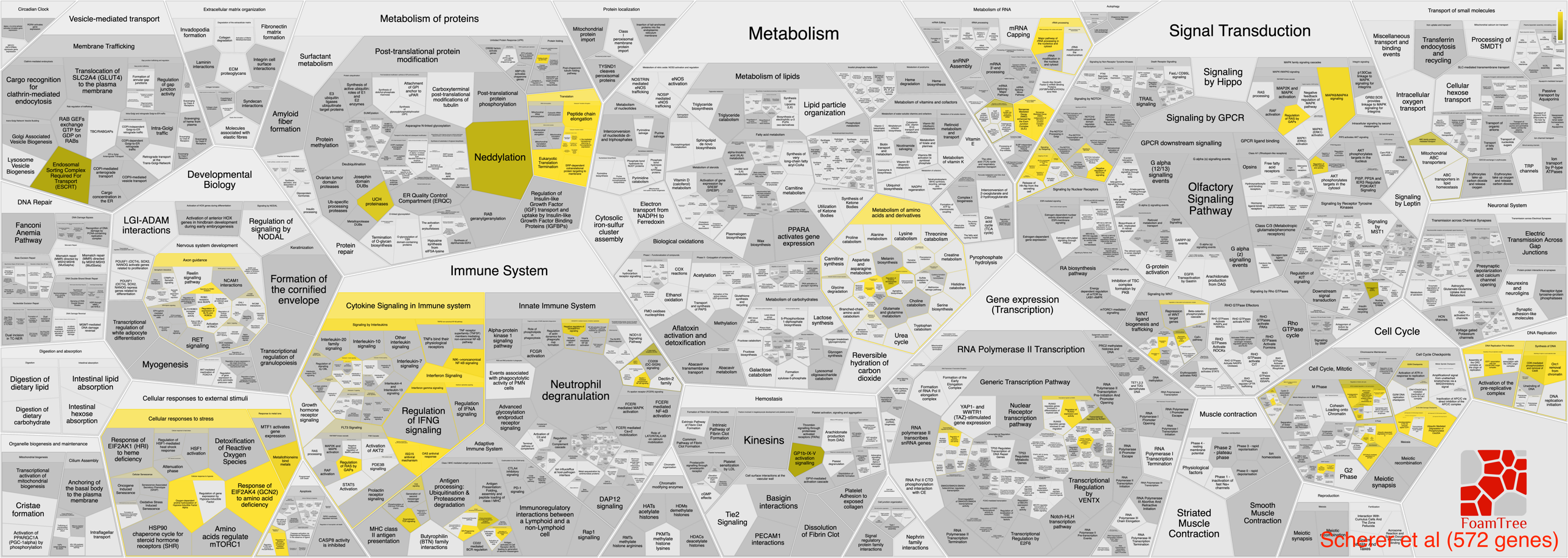

Supplement: Supplementary Figure 4 — More detailed results of the Reactome pathway enrichment analysis highlighting significant pathways induced by YF-Vax-induced immune factors as identified in Scherer et al. (14) (571 genes). The complexity of biomolecular pathways is organized by hierarchical groupings of the FoamTree (https://carrotsearch.com/foamtree/) and the color gradient highlights most to least significant pathways (p-value < 0.05). [file Image_4.PNG]
